# Supplementary material for: Factors influencing in-service teachers’ technology integration model: Innovative strategies for educational technology
Source: PLoS One. 2023 Aug 24;18(8):e0286112. doi: 10.1371/journal.pone.0286112 (PMC10449176; doi:10.1371/journal.pone.0286112)
Supplement: Attachment — Submitted filename: Rebuttal letter.doc [file pone.0286112.s002.doc]

**Rebuttal letter**

Dear Editor, Dear reviewers

Thank you for your letter. We are pleased to know that our rated as potential acceptable for publication in journal, subject to adequate revision. We thank the reviewers for the time and effort that they have put into reviewing the previous version of the manuscript. Their suggestions have enabled us to improve our work. Based on the instructions provided in your letter, we uploaded the file of the revised manuscript. Accordingly, we have uploaded a copy of the original manuscript with all the changes labeled by using different colors.

The revised paragraph based on Reviewer 1 is labeled in green.

The revised paragraph based on Reviewer 2 is labeled in blue.

Appended to this letter is our point-by-point response to the comments raised by the reviewers and editor. The comments are reproduced, and our responses are given directly afterward in a different color (red).

We would like also to thank you for allowing us to resubmit a revised copy of the manuscript.

We hope that the revised manuscript is accepted for publication in the journal of *PLOS ONE.*

Sincerely.

**Response to Reviewer 1 Comments**

**Point 1:** The authors have given their declaration that they can send all the data they used while reaching the results of their research upon request. However, it would have been easier to enter data into a database and download it from there instead.

**Response 1**: Thank you for underling this deficiency. We have updated the data in the supporting information, S1, data.

Special thanks to you for your good comments.

**Response to Reviewer 2 Comments**
**Point 1:** Please add the more latest citation in the paper.

**Response 1**: Thank you for your suggestion. We have added the latest citation in the paper as seen in references [6], [8], [9], [26], [30], [33], [36],[39], [40], [44], [49],[51], [56], [60] (Line 556-817, Page 34-40).

**Point 2:** Compared the discussion with latest exiting literature.

**Response 2:** Thank you for your suggestion, we have compared the discussion with latest exiting literature [26], [30], [33], [36],[39], [40], [44], [49],[51], [56], [60] (Line 556-817, Page 34-40).

**Point 3:** In Detailed, please explained the Research gap.

**Response 3**: Thank you for your suggestion. We have explained the research gap in the manuscript (Line 69-76, Page 4).

**Point 4:** Please Add the research hypothesis and Research Question.

**Response 4**: Thank you for your suggestion. We have added research questions and research hypotheses have listed in literature review part (Line 77-83, Page 4).

**Point 5:** Check the style of the reference according to the requirement of the journal.

**Response 5:** Thank you for underling this deficiency. We have modified the style of the reference according to the requirement of the journal (Line 556-817, 34-40).

Special thanks to you for your good comments.
